# Hydroxy-α sanshool induces colonic motor activity in rat proximal colon: a possible involvement of KCNK9

**DOI:** 10.1152/ajpgi.00114.2014

**Published:** 2015-01-29

**Authors:** Kunitsugu Kubota, Nobuhiro Ohtake, Katsuya Ohbuchi, Akihito Mase, Sachiko Imamura, Yuka Sudo, Kanako Miyano, Masahiro Yamamoto, Toru Kono, Yasuhito Uezono

**Affiliations:** ^1^Tsumura Research Laboratories, Tsumura & Co., Ibaraki, Japan;; ^2^Division of Cancer Pathophysiology, National Cancer Center Research Institute, Tokyo, Japan;; ^3^Department of Gastroenterology, Hokkaido University Graduate School of Medicine, Sapporo, Japan; and; ^4^Center for Clinical and Biomedical Research, Sapporo Higashi Tokushukai Hospital, Sapporo, Japan

**Keywords:** hydroxy-α sanshool, two-pore domain potassium channels, KCNK9, KCNK3, colonic motility, long distance contraction, rhythmic propulsive motor complex, rhythmic propagating ripple, postoperative ileus

## Abstract

Various colonic motor activities are thought to mediate propulsion and mixing/absorption of colonic content. The Japanese traditional medicine daikenchuto (TU-100), which is widely used for postoperative ileus in Japan, accelerates colonic emptying in healthy humans. Hydroxy-α sanshool (HAS), a readily absorbable active ingredient of TU-100 and a KCNK3/KCNK9/KCNK18 blocker as well as TRPV1/TRPA1 agonist, has been investigated for its effects on colonic motility. Motility was evaluated by intraluminal pressure and video imaging of rat proximal colons in an organ bath. Distribution of KCNKs was investigated by RT-PCR, in situ hybridization, and immunohistochemistry. Current and membrane potential were evaluated with use of recombinant KCNK3- or KCNK9-expressing *Xenopus* oocytes and Chinese hamster ovary cells. Defecation frequency in rats was measured. HAS dose dependently induced strong propulsive “squeezing” motility, presumably as long-distance contraction (LDC). TRPV1/TRPA1 agonists induced different motility patterns. The effect of HAS was unaltered by TRPV1/TRPA1 antagonists and desensitization. Lidocaine (a nonselective KCNK blocker) and hydroxy-β sanshool (a geometrical isomer of HAS and KCNK3 blocker) also induced colonic motility as a rhythmic propagating ripple (RPR) and a LDC-like motion, respectively. HAS-induced “LDC,” but not lidocaine-induced “RPR,” was abrogated by a neuroleptic agent tetrodotoxin. KCNK3 and KCNK9 were located mainly in longitudinal smooth muscle cells and in neural cells in the myenteric plexus, respectively. Administration of HAS or TU-100 increased defecation frequency in normal and laparotomy rats. HAS may evoke strong LDC possibly via blockage of the neural KCNK9 channel in the colonic myenteric plexus.

control of the mechanical activity of the intestines is complex and our understanding of the mechanisms involved is still in its infancy. Elements of the regulation of motor activity and the electrical and mechanical properties of the intestines have been found to be different depending on the species, regions of intestines under investigation, conditions of the specimen (in vivo or in vitro, unstimulated or stimulated, etc.), and methodology used for evaluation. In the rat colon, several distinct motor patterns have been demonstrated by Huizinga and colleagues (3, 11). Among them, the patterns termed “rhythmic propulsive motor complex (RPMC)/long distance contraction (LDC)” and “rhythmic propagating ripple (RPR)” have been proposed to be created, at least partly, by two independent networks of interstitial cells of Cajal (ICC). It has been suggested that RPMC/LDC and RPR may be related to the propulsion and mixing/absorption of the luminal contents, respectively. Therefore, agents affecting these motor patterns could lead to the development of new therapeutic options for colonic dismotility diseases such as constipation.

TU-100, a pharmaceutical-grade traditional Japanese (kampo) medicine, has been widely used for the treatment of various gastrointestinal (GI) disorders including postoperative ileus and ischemic intestinal disorders (17). The drug has been approved as a prescription drug by the Ministry of Health, Labor and Welfare of Japan and integrated into the modern medical care system in Japan. A double-blind, placebo-controlled study on healthy volunteers in the U.S. has shown that treatment with TU-100 significantly accelerates ascending colon emptying (22). Several double-blind placebo-controlled trials on the patients with postoperative ileus (POI), Crohn's disease, functional constipation, and irritable bowel syndromes are currently underway in the U.S. and Japan. The drug is an extract powder made from a mixture of Japanese pepper, processed ginger, and ginseng radix, with maltose powder as an additive. Hydroxy-α sanshool (HAS) contained in Japanese pepper has been elucidated as one of the main active compounds responsible for the efficacy of TU-100 to POI and adhesive intestinal obstruction (31, 32). Furthermore, HAS is rapidly absorbed in the gut and reaches high concentrations in the blood when TU-100 is administered orally (12, 24, 25). Hydroxy-β sanshool (HBS), a geometrical isomer of HAS, is also rapidly absorbed into the bloodstream (12, 24, 25). HAS and HBS, which have been known as agonists to transient receptor potential vanilloid type 1 (TRPV1) and transient receptor potential ankyrin 1 (TRPA1) (18), are now recognized as selective blockers to certain two-pore domain potassium (KCNK) channels: HAS for TASK-1 (KCNK3), TASK-3 (KCNK9), and TRESK (KCNK18), and HBS for KCNK3 (1).

TRPA1 expresses abundantly in enterochromaffin cells and TRPA1 stimulation induces serotonin (5-HT) release, resulting in enteric nerve activation (26), which may be a trigger for stimulus-induced colonic motility (10). KCNK3 has been reported to express in colonic smooth muscle cells (SMC) and to be involved in the determination of the resting potential (and therefore excitability) of SMC, which may affect the contractility of colonic smooth muscle (28). In the present study, we characterized the motility induced by HAS and investigated the mechanism of motility focusing on the possible involvement of TRP and/or KCNK channels.

## MATERIALS AND METHODS

### 

#### Chemicals and drugs.

HAS, HBS, TU-100, and maltose were supplied by Tsumura. Capsaicin (Sigma-Aldrich, St. Louis, MO), allyl isothiocyanate (AITC; Tokyo Chemical Industry, Tokyo, Japan), BCTC (COSMO BIO, Tokyo, Japan), HC-03003 (COSMO BIO), and lidocaine (Lid; MP Biomedicals, Santa Ana, CA) were commercially obtained. Other chemicals were purchased from Wako Pure Chemical Industries (Osaka, Japan).

#### Animals.

Male Sprague-Dawley rats (Japan SLC, Hamamatsu, Japan) at 7–12 wk old were housed under controlled-light environmental conditions and had free access to food and water. All experimental procedures were ethically approved by the Laboratory Animal Committee of Tsumura and Co. and performed according to the institutional guidelines for the care and use of laboratory animals, which is in accordance with the National Institutes of Health Guide for the Care and Use of Laboratory Animals.

#### Isolated rat proximal colon tract and measurement of intraluminal pressure.

Rats were fasted overnight and euthanized by decapitation before removing their entire colon. A 2- to 3-cm segment of the proximal colon was placed into an organ bath (100 ml volume), which was continuously perfused with warm Krebs solution (3.5 ml/min, 34–35°C). The oral and aboral ends of the proximal colon segment were securely attached with string to an input and output port of the saline, respectively. To monitor intraluminal pressure (cmH_2_O), a Mikro-Tip catheter pressure transducer (SPR-524, Millar Instruments, Houston, TX) was set in the lumen of the aboral end. Motility was initiated by loading an intraluminal pressure to ∼4 cmH_2_O by elevating the drain tube. After an equilibration period of 120–240 min, contraction had reached a consistent pattern, in terms of amplitude and frequency. The intraluminal pressure waves were evaluated by a data acquisition and analysis system (MP100, BIOPAC Systems, Goleta, CA). The motility was macroscopically observed through video images (PCR-SR87, SONY, Tokyo, Japan). All drug solutions were added to Krebs solution in the organ bath (serosal side). Peak frequency (PF) was calculated as the mean number of pressure peaks per minute during a defined period. The peak pressure amplitude (PPA) was calculated as the mean pressure of peaks during an allotted time period. The area under the curve (AUC) during the allotted time period was also calculated. Percentages of PF, PPA, and AUC were calculated for each period as the ratio to before drug treatment.

#### Spatiotemporal mapping.

**Fig. 1.**
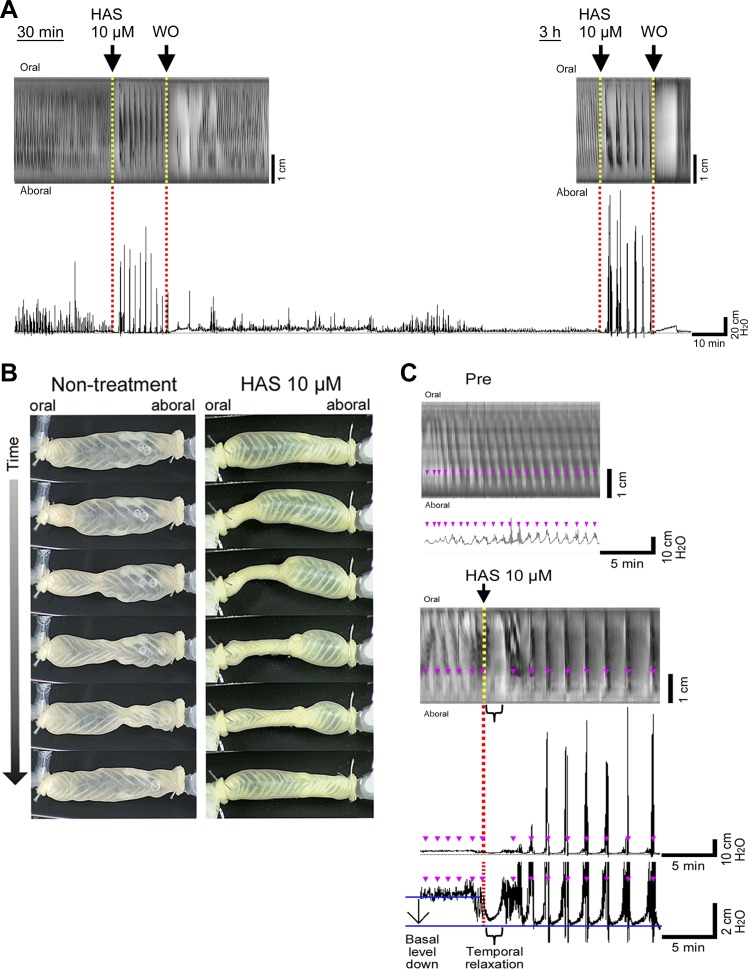
Induction of colonic motor activity and intraluminal high-amplitude pressure by hydroxy-α sanshool (HAS) in rat proximal colons. *A*: an entire recording of intraluminal pressure in a representative experiment is shown. A colonic specimen was placed in the bath and video images and intraluminal pressure were taken from *time 0* to 4 h. At 30 min, 10 μM of HAS was added, and at 0.75 h, HAS was washed (WO) out by several exchanges with fresh buffer. At 3 h, 10 μM of HAS was added again, and at 3.25 h, HAS was washed out. Moderate amplitude of pressure peaks continued until ∼2 h and thereafter only very small peaks were detected. HAS added at 30 min and 3 h gave essentially the same motor pattern with similar amplitudes and intervals. *B*: serial photographs of motility of nontreated (*left*) and HAS-treated (*right*) colons. Movie data are shown in Supplementary Video S3. *C*: relationship between the intraluminal pressure chart and spatiotemporal map. *Top*: no-treatment control (at 3 h) showing rhythmic propulsive motor complex (RPMC) with low amplitude. Pre means the motility before HAS addition. *Bottom*: HAS treatment (at 3 h). A representative example of long-distance contraction (LDC). Propulsive contractions are displayed as diagonal streaks of dark color. After the addition of HAS, a brief temporal relaxation is frequently observed in the pressure chart (see 10-fold magnified view), but this is always observed by video imaging. The relaxation response is indicated in the figure (labeled “Temporal relaxation”) with the change in minimum pressure levels (labeled by “Basal level down”) before and after HAS application. Nonpropulsive contractions are displayed as short, fragmented streaks of dark color. Pink dots in the spatiotemporal map correspond to those in the intraluminal pressure chart.

A spatiotemporal map, which is an image representation of motor activity, was generated according to the method described by Huizinga et al. (11). Colon width (coded as image intensity, black to white) is calculated at each point along the length of the colon (image *y*-axis), for each video frame (image *x*-axis) using ImageJ software. As shown in Fig. 1*C*, propagating contractions are represented as the diagonal streaks of dark color. Broad relaxation is represented as white area.

#### RT-PCR.

Muscle layer was carefully detached from the rat ascending colon and the remaining layer was used as the mucosal layer. Total RNA was prepared with RNeasy Universal Tissue Kit (Qiagen, Hilden, Germany) and cDNA was synthesized with High Capacity cDNA Reverse Transcription Kits (Life Technologies) according to the manufacturer's instructions. Sequences of sense and anti-sense primers for RT-PCR analysis were as follows: rat KCNK3 sense 5′-TCATCACCACAATCGGCTAT-3′, anti-sense 5′-AGCGCGTAGAACATGCAGAA-3′; rat KCNK9 sense 5′-CCTTCTACTTCGCTATCAC-3′, anti-sense 5′-CCAGCGTCAGAGGGATAC-3′; KCNK18 sense 5′-CTCACTTCTTCCTCTTCTTCTC-3′, anti-sense 5′-TAGCAAGGTAGCGAAACCTCT-3′; and GAPDH sense 5′-CGCATCTTCTTGTGCAGT-3′, anti-sense 5′-AATGAAGGGGTCGTTGATGG-3′. An aliquot of the RT reaction product served as a template in 30 cycles with 10 s of denaturation at 98°C, 30 s of annealing at 55°C, and 20 s of extension at 68°C by using the DNA polymerase KOD FX (TOYOBO, Osaka, Japan). A portion of the PCR mixture was electrophoresed through a 2% agarose gel in Tris-acetate-EDTA buffer (pH 8.0), and the gel was stained with ethidium bromide and imaged on a Typhoon 9410 imager (GE Healthcare, Piscataway, NJ).

#### In situ hybridization.

In situ hybridization (ISH) was performed by using QuantiGene ViewRNA. Gene specific probe sets for rat KCNK3, KCNK9, and PGP9.5 mRNAs were designed by Affymetrix (Santa Clara, CA). Muscle layer was collected from rat ascending colon. Fixation and hybridization were conducted by GeneticLab (Sapporo, Japan). Hybridized target mRNAs were visualized by bright-field microscopy (BIOREV BZ-9000, Keyence, Osaka, Japan).

#### Immunohistochemistry.

Whole-mount immunohistochemistry was performed as follows: Intestinal specimens were opened along the mesenteric border. The specimens were stretched taut and pinned out flat to a silicone ring and fixed with ice-cold acetone for 30 min. After fixation, each preparation was washed three times for 10 min each in phosphate-buffered saline (PBS; 0.9% NaCl in 0.1 M sodium phosphate buffer, pH 7.0). The preparations were placed in Superblock (Thermo Fischer Scientific, Rockford, IL) containing 0.3% Triton X-100 overnight at 4°C. The preparations were then placed in primary antibody diluted in antibody diluent (DAKO Japan, Tokyo, Japan) overnight at 4°C. After removal from the primary antibody, the tissues were rinsed for 3 × 10 min with PBS and incubated with the relevant secondary antibody conjugated to Alexa fluorochromes (Molecular Probes, Eugene, OR) diluted in antibody diluent (DAKO Japan) overnight at 4°C. After a final set of rinses, the preparations were mounted on microslides and coverslipped with Prolong Gold antifade reagent (Molecular Probes). The slides prepared from whole-mount or 7-μm-thick sliced specimens were observed using confocal laser microscopy FV-100D (Olympus, Tokyo, Japan). The following antibodies were used: a mouse monoclonal antibody to KCNK9 (Sigma-Aldrich), a guinea pig polyclonal antibody to PGP9.5 (Abcam plc, Cambridge, UK), rabbit polyclonal antibodies to KCNK3 (Santa Cruz Biotechnology, Dallas, TX), CD117 (DAKO Japan), and platelet-derived growth factor receptor (PDGFR)-α (Santa Cruz Biotechnology); smooth muscle actin nuclei were visualized by staining with Alexa568-conjugated phalloidin and 4′,6-diamidino-2-phenylindole (DAPI), respectively (Molecular Probes). In double immunostaining, each cell was identified by a combination of DAPI and a respective marker, and double-positive and single-positive cells were counted by visual inspection.

#### Preparation of plasmids and cRNAs.

Rat KCNK3 cDNA was amplified from rat whole brain cDNA (Takara, Otsu, Japan) and introduced into pcDNA-DEST40 by a Gateway system (Life Technologies, Carlsbad, CA). Rat KCNK9 cDNA was purchased from OriGene (Rockville, MD). cRNAs were prepared with mMessage mMachine Kit (Life Technologies) according to the manufacturer's instructions.

#### Two-electrode voltage clamp assay.

Immature V and VI oocytes from *Xenopus* were enzymatically dissociated. Isolated oocytes were incubated at 18°C in ND-96 medium (96 mM NaCl, 2 mM KCl, 1.8 mM CaCl_2_, 1 mM MgCl_2_, 5 mM HEPES pH 7.4) containing 2.5 mM sodium pyruvate and 50 μg/ml gentamicin. Two-electrode voltage clamp assays were performed 2–4 days after cRNA injection. Currents were recorded with a GeneClamp 500 Amplifier (Molecular Probes, Sunnyvale, CA) and Digidata 1322A (Axon Instruments, New York, NY) and acquired by using pCLAMP (Molecular Devices) software.

#### Membrane potential assay.

Chinese hamster ovary (CHO)-K1 cells (ATCC, Manassas, VA) were seeded into 96-well plates (∼7,000 cells/well). Rat KCNK3 or KCNK9 were then transiently expressed in CHO-K1 with FuGENE HD Transfection Reagent (Promega, Madison, WI) according to the manufacturer's instructions. About 48 h after transfection, a membrane potential assay was performed with a FLIPR Membrane Potential Assay Kit (Molecular Devices). The change of membrane potential was monitored in terms of fluorescence intensity, measured with a FlexStation3 (Molecular Devices).

#### Analysis of defecation frequency.

In normal rats, HAS was orally administered and thereafter the rat's feces were counted cumulatively during a 5-h period under fasting. Laparotomy rats (7–8 wk old) were incised ∼4 cm in the median line of the abdomen after being anesthetized by intraperitoneal injection of Somnopentyl (Kyoritsu Seiyaku, Tokyo, Japan). The small intestine, cecum, and large intestine were exteriorized and covered with sterile gauze dampened with saline for 1 h being returned to the abdominal cavity, and the abdomen was closed. Rat feces were counted cumulatively over a 7-h period under the fasting state. Rats in the nonoperation group were only anesthetized. HAS and TU-100 were administered orally to rats by the gavage technique 3 or 4 h after abdominal closure.

#### Data analysis.

All values are expressed as means ± SE. Statistical significance between two groups was evaluated by *F*-test analysis of variance, followed by Student's *t*-test or Aspin-Welch's *t*-test. Statistical significance between three or more groups was evaluated by Dunnett's test. When the agents were added sequentially, repeated-measures ANOVA was used. A probability of less than 0.05 was considered statistically significant.

## RESULTS

### 

#### Evaluation of colonic motor activity by intraluminal pressure peaks and video imaging and effect of HAS.

Motility of isolated segments of the proximal colon was analyzed by the measurement of intraluminal pressure and video image recordings. During a 2-h period after the beginning of the experiment, moderate-amplitude pressure peaks were observed (see Fig. 1*A*). Thereafter the amplitude of these pressure peaks weakened and by the 3-h time point only very small peaks could be detected (see Fig. 1*A*, from *time 3 h* to *4 h*). Nonetheless, low-amplitude contractions were still detected by video imaging, which appeared to be propulsive (Fig. 1*A*; details shown in the spatiotemporal map of Fig. 1*C*) until the end of the experiment. These types of contractions do not always produce evident changes in pressure peaks. We performed quantitative analysis of colonic motility mainly by assessing changes in the pressure because they are in good agreement with the motility observed in video imaging, at least for the high-amplitude propagating motor activity. Relationship between the intraluminal pressure chart and the video images is shown in Supplementary Video S1 and S2 (Supplemental Material for this article is available on the Journal website), and the relationship between the intraluminal pressure chart and spatiotemporal map is shown in Fig. 1*C*. It should be noted that, for the low-amplitude motor activity, not all of the activity produces evident changes in the pressure peaks. Therefore, analyses based on the pressure peaks may tend to underestimate its frequency.

**Fig. 2.**
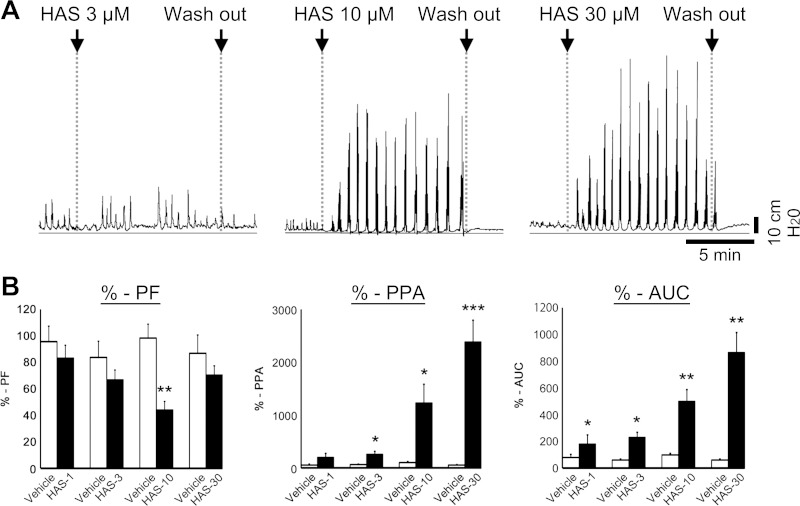
Dose-dependent induction of high-amplitude pressure peaks by HAS. *A*: typical patterns of intraluminal pressure induced by HAS. *B*: quantitation of HAS-induced contractility. Ratio of peak frequency (PF), peak pressure amplitude (PPA), and area under the curve (AUC) calculated as the ratio to the PF, PPA, and AUC before drug treatment (%-PF, %-PPA, and %-AUC, respectively) are shown (*n* = 4–6). *P < 0.05, **P < 0.01, ***P < 0.001 vs. Vehicle control.

As shown in Fig. 1*A*, addition of HAS to the bath solution from the serosal side at 30 min and 3 h induced high-amplitude periodic pressure peaks with similar frequency and potency. The motility is characterized by a strong squeezing over a broad range of the proximal colon (Fig. 1, *B* and *C*, movie files are shown in Supplementary Video S3), after a brief temporal relaxation (Fig. 1*C*). The dose dependency of pressure peak pattern, PF, PPA, and AUC are shown in Fig. 2.

#### Contraction induced by HAS is not a result of stimulation of TRPV1/TRPA1.

**Fig. 3.**
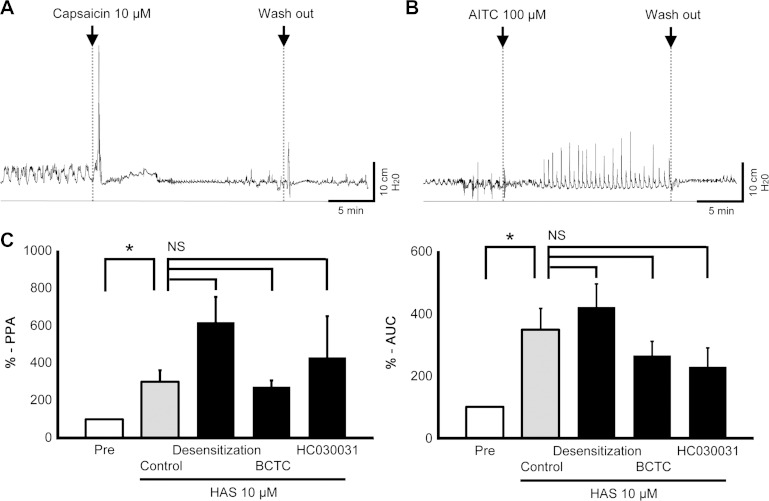
Motor activity by HAS is not mediated by TRPV1 nor TRPA1. Typical changes of intraluminal pressure induced by a TRPV1 agonist capsaicin (*A*) and a TRPA1 agonist allyl isothiocyanate (AITC, *B*). *C*: effects of desensitization (pretreatment with 3 μM capsaicin plus 10 μM AITC), a TRPV1 antagonist BCTC (1 μM), or a TRPA1 antagonist HC-030031 (5 μM) on HAS-induced motility (*n* = 5–6). Pre means the motility before HAS addition. *P < 0.05. NS, not significant.

Firstly, we investigated whether TRPV1/TRPA1 agonists evoke motility similar to that induced by HAS. A TRPV1 agonist capsaicin (0.1–10 μM) evoked a single contraction peak only, both at 30 min (data not shown) and 3 h (Fig. 3*A*; Supplemental Video S4) although its PPA was potent. A TRPA1 agonist AITC induced periodic contractions, although the amplitude was very small even at high concentrations (Fig. 3*B* represents the result of AITC added at 100 μM at 3 h; Supplementary Video S5). Furthermore, administration of TRPV1 or TRPA1 inhibitor (BCTC and HC-030031, respectively) and desensitization of TRPV1/TRPA1 by bolus application of high doses of capsaicin plus AITC, gave no, or only a modest, suppression of colonic motility evoked by HAS (Fig. 3*C*).

#### Localization of KCNK9 and KCNK3 proteins.

**Fig. 4.**
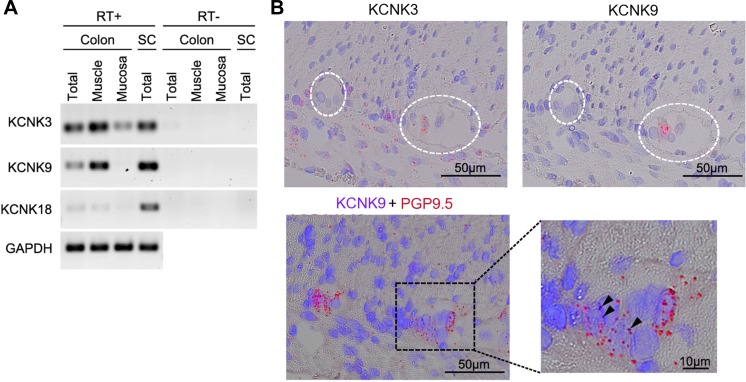
Distribution of mRNA of KCNK channels in rat colon. *A*: RT-PCR analysis of mRNA expression in rat colon. PCR products were electrophoresed through a 2% agarose gel. RT, reverse transcriptase, SC, spinal cord. *B*: in situ hybridization of rat colon for KCNK3, KCNK9, and PGP9.5. *Top*: single staining for KCNK3 or KCNK9. Red dots indicate the target mRNA. Location of the myenteric plexus is indicated by a dashed white circle. *Bottom*: double staining for PGP9.5 and KCNK9. Red and violet dots indicate PGP9.5 and KCNK9 mRNAs, respectively. KCNK9 mRNA is indicated by arrows. Nuclei were stained with hematoxylin.

**Fig. 5.**
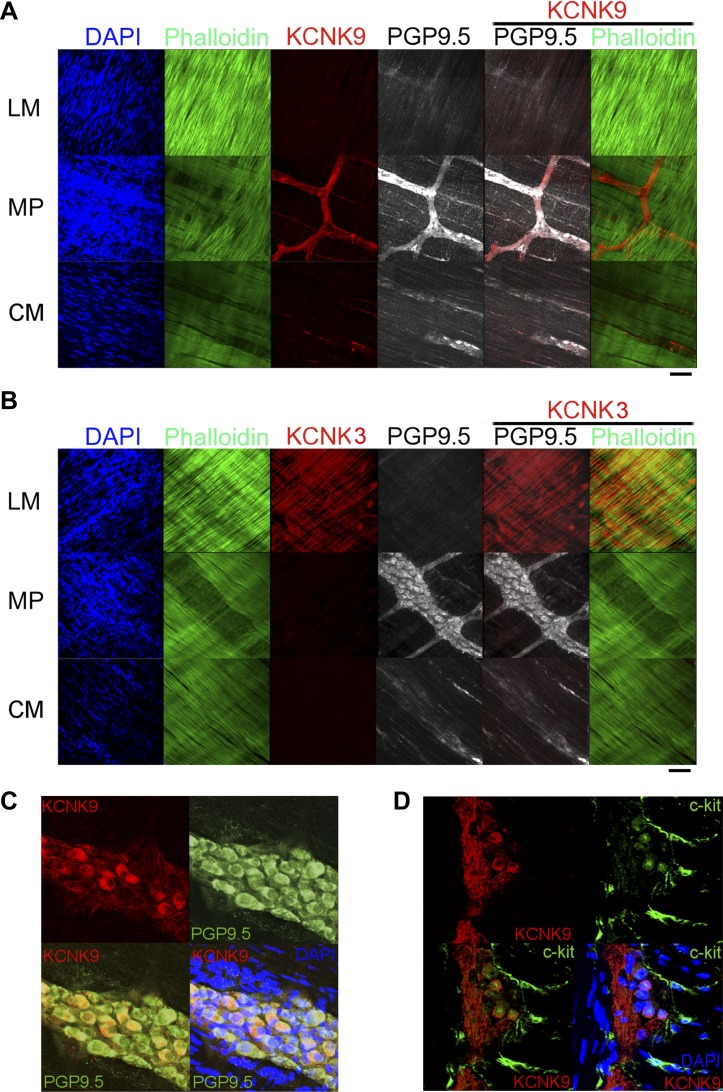
Distribution of KCNK9 and KCNK3 proteins in rat colons *A*: immunohistochemistry of whole-mount muscle layer of rat colon for KCNK9, phalloidin, and PGP9.5. DAPI, 4′,6-diamidino-2-phenylindole; LM, longitudinal muscle; MP, myenteric plexus; CM, circular muscle. *B*: immunohistochemistry of whole-mount muscle layer of rat colon for KCNK3, phalloidin, and PGP9.5. *C*: double immunostaining of KCNK9 and PGP9.5. in MP. *D*: double immunostaining of KCNK9 and c-Kit in MP. Scale bar = 30 μm.

To investigate the possible involvement of these KCNK channels in HAS-induced motility, RT-PCR was performed with specific primers for rat KCNK3, KCNK9, and KCNK18. Our results show that KCNK3 and KCNK9 mRNAs are expressed in the muscle layer, whereas KCNK18 mRNA is barely detectable in the rat proximal colon (Fig. 4*A*). The localization of KCNK3 and KCNK9 was evaluated by ISH and immunohistochemistry. As shown in Fig. 4*B*, KCNK3 mRNA was localized in the longitudinal muscle (LM) layer and myenteric plexus (MP) and KCNK9 mRNA in MP (Fig. 4*B*). A neuronal marker PGP9.5 mRNA coexisted with KCNK9 mRNA (Fig. 4*B*). Immunohistochemistry of whole-mount preparation of colonic muscle layers confirmed the predominant localization of KCNK9 in MP and in the enteric nerves in the circular muscle (CM) layer (Fig. 5*A*), and that of KCNK3 in LM SMCs (Fig. 5*B*). Coimmunostaining analysis with anti-KCNK9 and anti-PGP9.5 antibodies (Fig. 5*C*) showed that KCNK9 is expressed in 35.8% of PGP9.5^+^ MP cells (*n* = 148). More than a quarter of KCNK9^+^ cells in MP are PGP9.5^−^. Furthermore, in coimmunostaining experiments with KCNK9 and an ICC marker c-Kit (Fig. 5*D*), 22.4% of c-Kit^+^ cells in MP were found to be KCNK9^+^ (*n* = 156) though the double-positive cells had relatively weaker c-Kit immunosignals (i.e., most of the cells with the strongest c-Kit immunosignals lacked KCNK9 immunosignal). Nearly half of KCNK9^+^ cells in MP were c-Kit^−^ (often located adjacent to c-Kit^+^ cells). KCNK9 signals were not detected in PDGFR-α-positive fibroblast-like interstitial cells (data not shown), which are thought to participate in inhibitory neurotransmission of enteric nerves.

#### HAS induced membrane depolarization via blocking rat KCNK9.

**Fig. 6.**
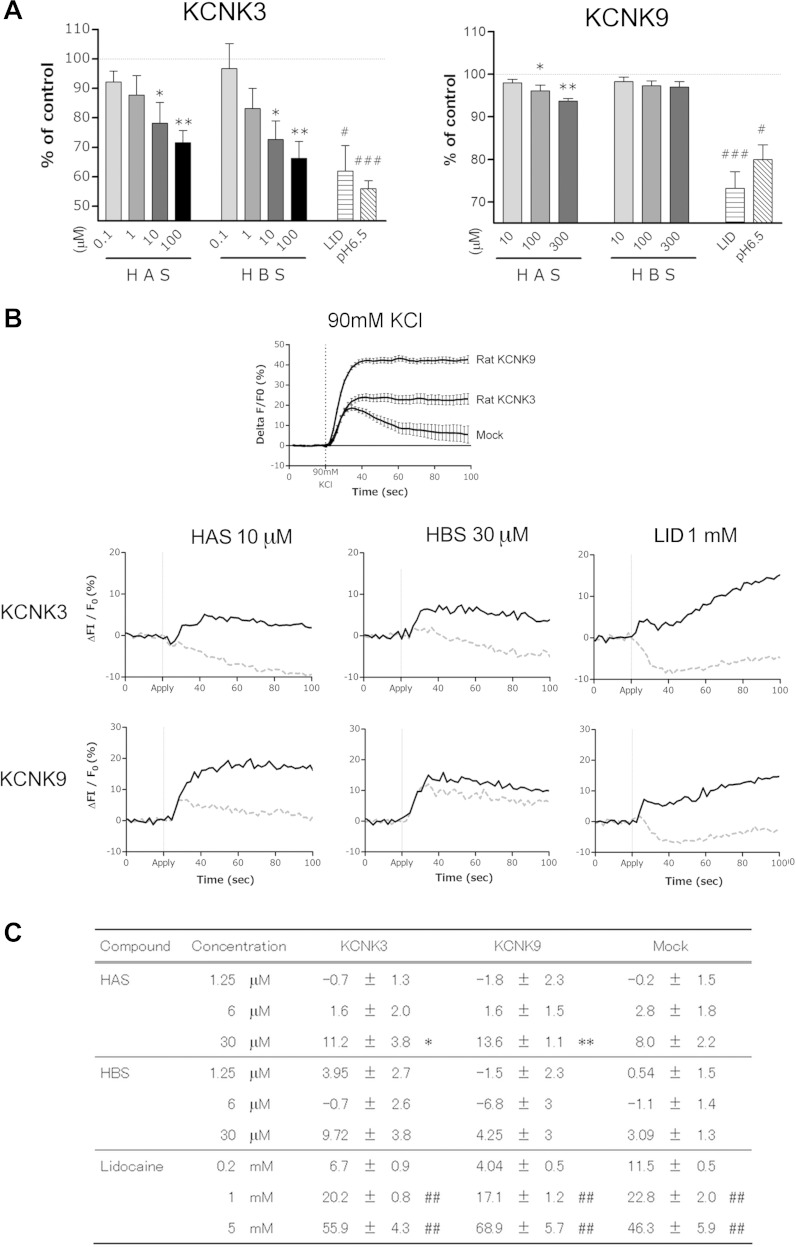
HAS induces depolarization by blocking KCNK3 and KCNK9. *A*: 2-electrode voltage-clamp analysis with *Xenopus* oocytes expressing rat KCNK3 or KCNK9. Percent suppression of leak current was determined with a holding potential of +60 mV. Data are expressed as means ± SE for *n* = 4–8. *P < 0.05, **P < 0.01 vs. DMSO control. #*P* < 0.05, ###*P* < 0.001 vs. PBS control. *B*: kinetics of the changes of membrane potential responses induced by HAS, hydroxy-β sanshool (HBS), and lidocaine (Lid) in rat KCNK3- or KCNK9-expressing CHO-K1 cells. Application of compounds is indicated by “Apply.” Kinetics of the changes in membrane potential responses induced by 90 mM KCl is included as a positive control (*n* = 4). *C*: summary of normalized response to KCNK3, KCNK9, and mock cell by HAS, HBS, and Lid. Values represent the ΔF/F_0_ normalized to that of DMSO or PBS controls (mean ± SE for *n* = 4). *P < 0.05, **P < 0.01, ##*P* < 0.01.

It is not known whether HAS and HBS inhibit rat KCNK3 and/or KCNK9. Thus we conducted two electrode voltage-clamp assays using *Xenopus* oocytes expressing rat KCNK3 or KCNK9. Because both channels were reported to be regulated by extracellular pH, application of pH 6.5 solution was used as a positive control. Inhibitory effect of test compounds were determined with a holding potential of +60 mV. Lid was used as a nonselective KCNK channel blocker, which strongly inhibited both KCNK3 and KCNK9. As shown in Fig. 6*A*, HAS showed significant and dose-dependent inhibition against KCNK3 and KCNK9 whereas HBS inhibited only KCNK3. These results were comparable to those observed for murine KCNKs (1).

KCNK channels regulate the excitability of cells such as smooth muscle and neurons by adjusting their membrane potential. To address whether HAS inhibition against KCNK3 and KCNK9 led to membrane depolarization, we examined the membrane potential of rat KCNK3- or KCNK9-expressing CHO-K1 cells (Fig. 6, *B* and *C*) by using a membrane potential dye. In this assay, the fluorescent signal increases during membrane depolarization and decreases during membrane hyperpolarization. For example, the application of KCl increased the fluorescent signal, which represents a depolarizing membrane potential. The KCl-induced changes in fluorescence intensity for KCNK3 and KCNK9 expressing cells were larger than that observed for the mock cells (Fig. 6*B*). Data are represented by the normalized change in fluorescence (ΔF/F_0_). Inhibition of KCNK3 or 9 was determined at 100 s after compound application. The application of HAS and Lid to KCNK3- and KCNK9-expressing cells significantly increased the fluorescence intensity. The effect induced by 30 μM of HAS was comparable to that induced by 1 mM of Lid, although Lid induced depolarization not only in KCNKs-expressing cells but also in mock cells (Fig. 6*C*). HBS appeared to induce depolarization of KCNK3-expressing cells but the effect was not significant (Fig. 6, *B* and *C*).

#### HBS and Lid induce colonic motor patterns different from HAS.

**Fig. 7.**
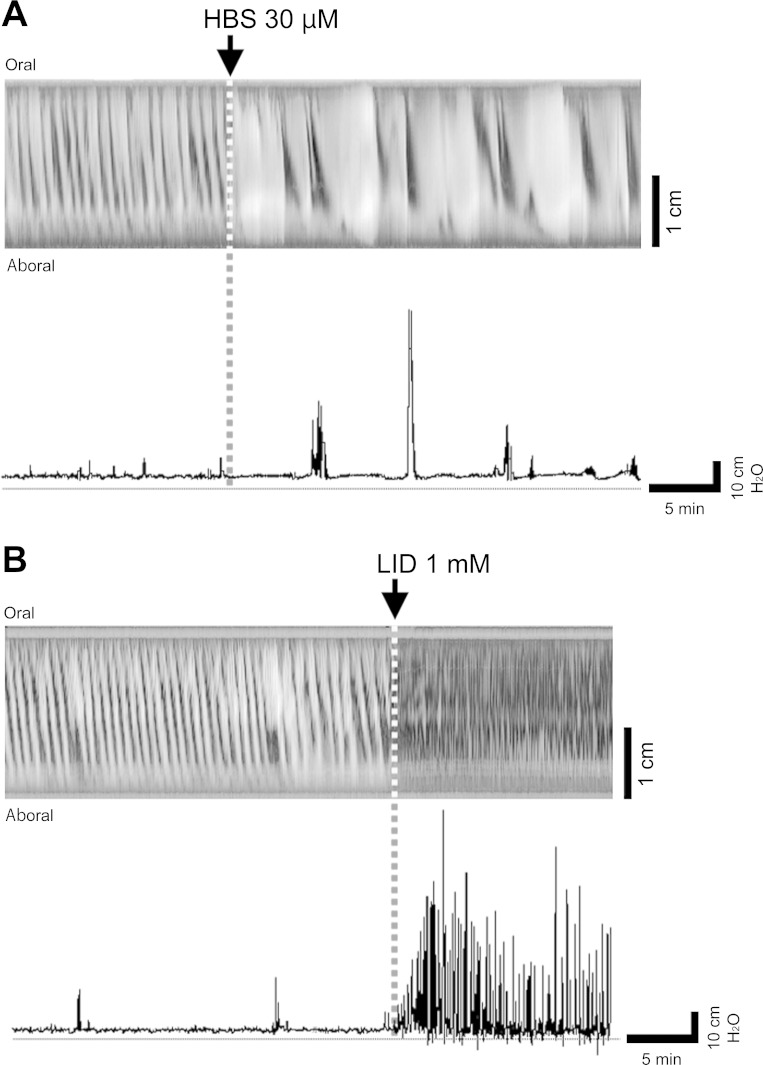
Motor activity induced by HBS and Lid. The typical patterns of HBS (*A*) and Lid (*B*) on a spatiotemporal map and intraluminal pressure chart are shown.

Although HAS, HBS, and Lid raised the intraluminal pressure of rat isolated colons and induced potent contraction/motility, the resulting motor patterns differed among these three compounds. Although the pressure peaks induced by HAS were strong and repeated periodically with almost the same frequency, those induced by HBS were lower in frequency and less periodic (Fig. 7*A*, Supplementary Video S6). The effects induced by Lid were also weaker in amplitude, not periodic, but higher in frequency than those induced by HAS (Fig. 7*B*, Supplementary Video S7). The spatiotemporal maps also suggested that HAS induces potent motor activity propagating from the oral side to the aboral side over the entire proximal colon (Fig. 1*A*), whereas Lid was found to induce high-frequency, low-amplitude, nonpropulsive motor activity (Fig. 7*B*). Table 1 shows the PF of HAS-, HBS-, and Lid-induced colonic motility.

**Table 1.** Peak frequency of HAS, HBS, and Lid

Agents	None	None	HAS (10 μM)	HBS (30 μM)	Lid (1 mM)
Measured time	0–0.25 h	3–3.25 h	3–3.25 h	3–3.25 h	3–3.25 h
Peak frequency, number/min	3.00 ± 0.41	1.27 ± 0.20	0.44 ± 0.03	0.21 ± 0.03	5.64 ± 0.66

The number of peaks during a set period of time was counted in the pressure peak chart and peak frequency (PF) was calculated as the number of peaks per minute.

HAS, hydroxy-α-sanshool; HBS, hydroxy-β-sanshool; Lid, lidocaine. *N* = 4–7.

#### High-amplitude contraction by HAS is nerve mediated.

**Fig. 8.**
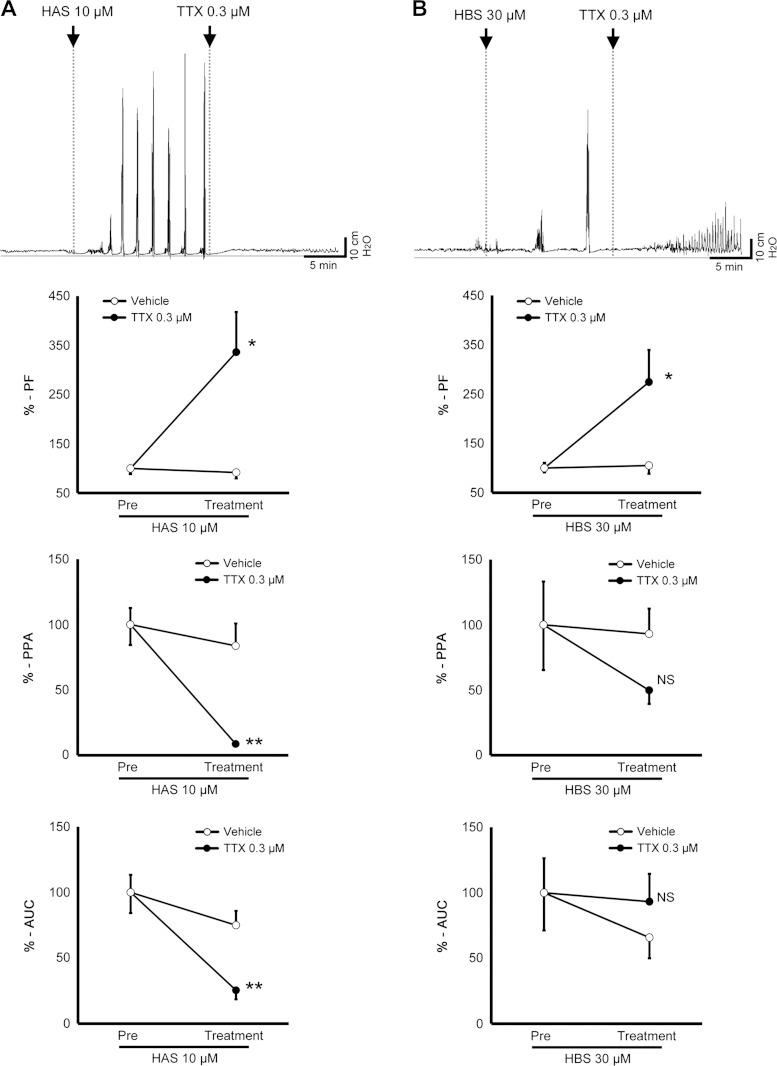
Effect of TTX on HAS- and HBS-induced contraction. *A* and *B*: *top* charts show typical changes in the HAS (*left*) and HBS (*right*) motility patterns induced by TTX. *Middle* and *bottom* graphs show changes of %-PF, %-PPA, and %-AUC induced by TTX, respectively. Experiment used 10 μM HAS (*n* = 6) and 30 μM HBS (*n* = 5 and 6). Pre means the motility before addition of TTX or its vehicle. *P < 0.05, **P < 0.01 vs. Control (vehicle).

The contractions induced by 10 μM HAS were suppressed almost completely by addition of TTX (0.3 μM), a neuroleptic agent (Fig. 8*A*). By contrast, the contractions induced by HBS were not abolished but its shape was changed by TTX (Fig. 8*B*). Addition of high doses (up to 3 mM) of TTX did not alter the effect of Lid (1 mM, data not shown).

#### HAS accelerates defecation in normal and POI model rats.

**Fig. 9.**
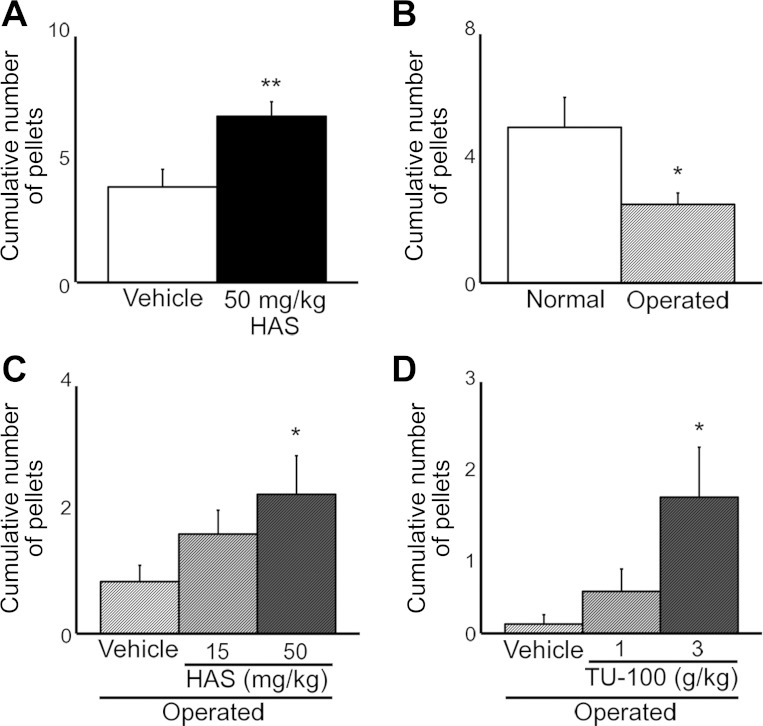
Acceleration of defecation by HAS and TU-100 in normal rats or postoperative ileus (POI) model rats. *A*: increase of defecation frequency at 5 h after HAS (50 mg/kg po) administration in normal rats. *P < 0.05, **P < 0.01 vs. Vehicle (olive oil 0.5 ml/kg po) (*n* = 8). *B*: decrease of defecation frequency by laparotomy. *P < 0.05, ***P < 0.001 vs. Normal (anesthetized only). (*n* = 8 ∼21, at 7 h after operation). *C*: increase of defecation frequency after HAS (15 and 50 mg/kg po) dosing at 4 h after administration (i.e., 6 h after operation) in POI rats. *P < 0.05 vs. Vehicle (*n* = 23–25). *D:* acceleration of defecation frequency induced TU-100 (1 and 3 g/kg po) dosing 3 h after administration (i.e., 6 h after operation) in POI rats. *P < 0.05, **P < 0.01 vs. Vehicle (water 15 ml/kg po) (*n* = 8 or 9).

To investigate whether HAS accelerates defecation in vivo we examined the amount of feces accumulated during a short period after treatment with the agents. The accumulated number of fecal pellets increased significantly at 5 h after oral administration of 50 mg/kg HAS to normal rats (Fig. 9*A*).

For rats that had undergone abdominal surgery, the number of pellets decreased significantly 7 h after suturing (Fig. 9*B*). Oral administration of HAS at 3 h after the operation significantly increased the number of pellets 4 h after administration (i.e., 7 h after the operation) in a dose-dependent manner (Fig. 9*C*). Accordingly, a single oral administration of TU-100 3 h after the operation also increased the number of pellets 3 h after drug treatment (i.e., 6 h after the operation) in a dose-dependent manner. A significant outcome was obtained at a dose of 3 g/kg TU-100 (Fig. 9*D*).

## DISCUSSION

HAS was found to induce high amplitude of contractions with significant levels of periodicity. The peak frequency (∼0.5/min), propagating/propulsive property, sensitivity to TTX, and the presence of relaxation phase preceding a very strong contractions are in good agreement with those reported for LDC (11). Lid, however, was found to induce low-amplitude contractions with high frequency (∼6/min), which were nonpropulsive and insensitive to TTX [i.e., in good agreement with RPR (11)]. The increase in amplitude induced by HBS might be periodic, but the intervals between consecutive peaks were wider and the incidence of the peaks appeared more irregular compared with those induced by HAS. As shown in Fig. 3, LDC induced by HAS was not caused by the agonistic activity of TRPV1/TRPA1, which is well documented for HAS and HBS. We reasoned that the target molecules of HAS, HBS, and Lid may be KCNK channels because these agents, especially HAS and HBS, have a high specificity for KCNK3 and/or KCNK9. Bautista et al. (1) have screened various ion channels including KCNK1∼6, 9, 10, 13, 16, and 18 and reported specific inhibition of KCNK3 by HBS and of KCNK3/9/18 by HAS. Lid blocks a broad range of KCNKs. Although many of the biological activities of Lid can be attributed to its inhibitory activity against multiple voltage-gated sodium channels, the effect on KCNK3 has been suggested to be involved in Lid-induced seizure in mice (6).

KCNK ion channels comprise a family of potassium-selective channels that share the unique structural feature of four transmembrane domains and two pore-forming domains (8). The KCNK channel family are open across the physiological voltage range and are therefore believed to underlie many of the background K^+^ currents (also known as resting, baseline, or leak currents) that regulate the resting membrane potential and excitability of many mammalian cells (2, 7, 21). KCNK channels are found in neuronal and nonneuronal tissues, and they provide, in addition to setting of resting membrane potentials (13, 23), a wide variety of important functions, including sensing of oxygen and pH, neuroprotection, and mechanosensitivity (28). These channels are also candidates for the action of volatile anesthetics on neural excitability (27). Molecular analysis of GI smooth muscle myocytes revealed expression of KCNK genes, including KCNK2, KCNK10 (14), KCNK3, and KCNK5 (28) and the properties of the currents produced by these channels have been shown to contribute to the native currents observed in GI smooth muscle (4). These data suggest that certain KCNK family channels may be involved in the regulation of GI motility.

Interestingly, we found that the KCNK9/KCNK3 blocker HAS evokes a unique motor pattern with a high degree of periodicity, whereas its geometric isomer, HBS, specifically blocked KCNK3 and induced a motor pattern different from that of HAS. This observation suggests that the highly periodic LDC evoked by HAS may be mediated by KCNK9. In the present study, we show that KCNK3 is located in LM SMC and presumably a small portion of neuronal cells in MP, whereas KCNK9 is located mainly in a portion of neuronal cells in MP and CM layer. These findings are in good accordance with previous immunohistochemical studies using mouse (14, 28) and human (19) GI tissue. The location of KCNK9 suggests that the channel may be involved in the determination of resting potential and excitability of a substantial portion of enteric neurons innervating the MP and CM layer. The LDC induced by HAS was completely abrogated by TTX treatment. If HAS blocks KCNK9 in these motor neurons, their excitability will be augmented. Under such conditions, even if signals with similar intensity are provided from upstream neurons (e.g., interneurons and intrinsic primary afferent neurons) and/or enteroendocrine cells, the strength of the signals to the ICC/SMC will differ substantially. Furthermore, although our data indicates that KCNK9 is not expressed in SMC, it addresses the possibility of its expression in ICC in MP (ICC-MP). ICC-MP network is a pacemaker and has been suggested to actively propagate rhythmic transient depolarizations responsible for RPMC and/or LDC (11, 29). If HAS affects ICC-MP to augment its functions, (i.e., to generate more intense and well-regulated electrical periodicity), the agent would evoke potent and regular LDC as demonstrated in the present results.

Because KCNK3 exists predominantly in LM SMC, it is thought to be involved in the regulation of excitability of SMC, which may chiefly affect the tone of muscle contractility. The present study indicates that KCNK3 might be expressed in some neurons in MP. This finding is intriguing because the effect of HBS, the blocker of KCN3, was not abrogated but its potency and frequency were changed by TTX, suggesting a possible involvement of motility control system of both neurogenic and myogenic origin. Chen et al. (3) have demonstrated that myogenic mechanisms could be involved in certain LDC-like rhythmic activity. Thus further detailed examination is necessary to clarify the effect of KCNK3 on colonic motility.

The properties of the Lid-induced motility are in good accordance with those of RPR, which is thought to be mediated by the submuscular ICC (ICC-SMP) network (11). Lid is primarily a potent inhibitor of several voltage-gated sodium channels and has a wide array of biological activities including the effects on other KCNK family channels (5, 20) in which the blocking of KCNK3 is only one item of the array. Accordingly, 1 mM Lid significantly increased the membrane potential in KCNK3-, KCNK9-, and mock-transfected CHO-K1 cells, which strongly suggests that the depolarization by Lid may be due to a mechanism other than its inhibition of KCNK3/9.

HAS is a major ingredient of TU-100, which has been integrated into the modern medical system under the approval of the Ministry of Health, Labor and Welfare of Japan. Basic research has revealed the various beneficial effects of TU-100 on intestinal motility (9, 30, 32), adhesion (31), vasodilatation (16), and inflammation (15). Clinical trials of TU-100 are currently underway in the U.S. aimed at the development of novel therapeutic treatments for various intestinal disorders. These studies include research on GI and colonic transit by validated scintigraphy, which has indicated that TU-100 significantly accelerates ascending colon empting in healthy human volunteers (22). Pharmacokinetics studies have shown that, when TU-100 is administered orally, HAS is rapidly absorbed in the gut and reaches high concentrations in the blood (∼1 μM in human and rats) within 15 min (12, 24, 25). The present experimental settings for the bath application of HAS to the proximal colon is in reasonable agreement with the clinical situation of orally administered TU-100 in terms of the pharmacological and pharmacokinetic properties of the drug. Furthermore, we have observed that low dosages of HAS, which are too low to induce LDC alone, enhance motility triggered by other stimuli such as bethanechol, capsaicin, or gingerol (data not shown). This observation is in good accordance with the assumed mechanism of action of HAS, i.e., augmentation of excitability of enteric motor neurons via blocking KCNK9.

To investigate whether the induction/enhancement of LDC in vitro relates to the colonic transit in vivo, we have examined the effect of HAS or TU-100 on defecation of normal and postoperative rats. A single administration of HAS or TU-100 increased the number of fecal pellets accumulated over a short period of time (3–4 h after drug treatment and 6–7 h after surgery).

Although the present study may provide potentially interesting findings, there are several points to be validated and investigated in future studies. Firstly, involvement of KCNK channels in HAS/HBS/Lid-mediated motility is still unclear. The use of more specific agonists and/or antagonists, and gene-manipulated mice is necessary to clarify this point. Secondly, further investigation into the biological function of KCNK channels in the generation of slow wave activity, rhythmic transient depolarizations, and colonic motility is needed. In the present study, we have demonstrated that KCNK channels strongly affect the membrane potential in the cell line. However, detailed analyses on GI cells and tissues, such as the measurement of inhibitory junction potential, have yet to be performed. Thirdly, whether the KCNK9 channel is expressed in ICC-MP should be determined by more conclusive and quantitative methods such as more extensive morphometrical immunohistochemistry of the tissues and analysis of KCNK9 protein and mRNA in purified ICCs. If KCNK9 is expressed in a subpopulation of ICC-MP, it will be reasonable for HAS to modulate the pace-making of colonic motility. Elucidation of these points will undoubtedly contribute to a deeper understanding of the physiology and molecular biology of colonic motor activities.

In conclusion, we have demonstrated that HAS may induce or enhance LDC of the proximal colon, presumably elevating the excitability of enteric nerves via KCNK9 blocking. The present findings provide not only a clarification of the mechanism of action of a promising new medicine TU-100 but also a way to develop a novel therapeutic strategy for the treatment of intestinal dysmotility.

## GRANTS

This project has been executed by using the institutions' (National Cancer Center Research Institute and Tsumura & Co.) budgets including a grant from Tsumura & Co. providing to T. Kono, Y. Sudo, K. Miyano, and Y. Uezono for this collaborative research.

## DISCLOSURES

K. Kubota, N. Ohtake, K. Ohbuchi, A. Mase, S. Imamura, and M. Yamamoto are employed by Tsumura & Co. Y. Uezono and T. Kono have financial interests from Tsumura & Co. relevant to this research. Y. Sudo and K. Miyano have an indirect financial interest from Tsumura & Co. relevant to this research.

## AUTHOR CONTRIBUTIONS

K.K., N.O., K.O., A.M., Y.S., K.M., M.Y., T.K., and Y.U. conception and design of research; K.K., N.O., K.O., A.M., S.I., and M.Y. performed experiments; K.K., N.O., K.O., A.M., and M.Y. analyzed data; K.K., N.O., K.O., A.M., Y.S., K.M., M.Y., T.K., and Y.U. interpreted results of experiments; K.K., N.O., K.O., A.M., and M.Y. prepared figures; K.K., K.O., and M.Y. drafted manuscript; M.Y., T.K., and Y.U. edited and revised manuscript; Y.U. approved final version of manuscript.

## Supplementary Material

Video Legends

Video S1

Video S2

Video S3

Video S4

Video S5

Video S6

Video S7
