# Supplementary material for: Hydroxy-α sanshool induces colonic motor activity in rat proximal colon: a possible involvement of KCNK9
Source: Am J Physiol Gastrointest Liver Physiol. 2015 Jan 29;308(7):G579–90. doi: 10.1152/ajpgi.00114.2014 (PMC4385894; doi:10.1152/ajpgi.00114.2014)
Supplement: Video Legends [file ad97733fa7f6658ad4da8945b5880252_video-legends.pdf]

Supplementary Movie Legends

Supplementary Movie M1. Relationship between the video image and intraluminal pressure peaks (non-stimulated).

Supplementary Movie M2. Relationship between the video image and intraluminal pressure peaks (bethanecol stimulated).

Supplementary Movie M3. Effect of 10 μmol/L HAS

Supplementary Movie M4. Effect of 10 μmol/L Capsaicin

Supplementary Movie M5.: Effect of 100 μmol/L AITC

Supplementary Movie M6. Effect of 30 μmol/L HBS

Supplementary Movie M7. Effect of 1 mmol/L LID
